# How teacher support and practical environment relate to engagement in project-based learning: the mediating roles of self-efficacy and autonomous motivation

**DOI:** 10.3389/fpsyg.2026.1958111

**Published:** 2026-09-14

**Authors:** Sunfa Liu, Bingli Gong

**Affiliations:** 1 Network Technology Center, Sanming University, Sanming, China; 2 Office of Educational Administration, Sanming University, Sanming, China

**Keywords:** autonomous motivation, PLS-SEM, practical environment and support, project-based learning, self-efficacy, student engagement, teacher behavior and support

## Abstract

**Background:**

Project-based learning (PBL) has been widely adopted in higher education, including Chinese universities, to promote active, interdisciplinary, and applied learning. Yet sustaining deep student engagement in demanding project environments remains a challenge, and existing research has largely examined instructional, environmental, and individual factors in isolation rather than within an integrated framework. Drawing on self-determination theory and social cognitive theory, this study distinguished two forms of contextual support, namely teacher behavior and support (TBS) and practical environment and support (PES), and examined how they relate to student engagement through self-efficacy (SE) and autonomous motivation (AM).

**Methods:**

A cross-sectional survey of 511 undergraduates enrolled in interdisciplinary PBL courses at a university in eastern China was analyzed using partial least squares structural equation modeling (SmartPLS 4), following a two-step procedure with 5,000 bootstrap resamples and blindfolding for predictive relevance. Common method bias was assessed through Harman's single-factor test, a full collinearity assessment, and a marker-variable adjustment.

**Results:**

The measurement model demonstrated satisfactory reliability, convergent validity, and discriminant validity. The structural model explained 56.8% of the variance in student engagement and showed adequate predictive relevance (*Q*^2^ > 0.28 for all endogenous constructs). TBS and PES had significant direct effects on engagement and significantly predicted SE and AM. Both SE and AM, in turn, significantly predicted engagement. Bootstrapped mediation tests confirmed four significant indirect pathways, indicating partial mediation. Notably, PES had large effects on SE (*f*^2^ = 0.476) and AM (*f*^2^ = 0.424), while SE showed a numerically larger coefficient than AM in predicting engagement (β = 0.307 vs. 0.175), although the difference did not reach statistical significance.

**Conclusion:**

These findings suggest that PBL engagement is associated with dual pathways: contextual support relates to students' confidence and their autonomous motivation, which together relate to sustained engagement in project work. By integrating the two theoretical frameworks and distinguishing interpersonal from structural support, the study highlights self-efficacy as a psychological resource that appears comparatively central in complex project environments, informing the design of more effective PBL environments.

## 1 Introduction

Project-based learning (PBL) has become an important pedagogical approach in higher education because it situates students in authentic, complex, and collaborative learning contexts. Rather than receiving knowledge passively, students define problems, integrate knowledge across disciplinary boundaries, negotiate solutions with peers, and produce outcomes with practical relevance. Prior reviews and meta-analyses demonstrate that PBL supports learning outcomes, problem solving, and higher-order thinking when it is carefully designed and adequately supported (Blumenfeld et al., 1991; Zhang and Ma, 2023). Recent research in Frontiers in Psychology has continued to position PBL, self-efficacy, and engagement as critical issues in higher education and applied learning contexts (Guan et al., 2026; Wang and Zhang, 2024).

Research has identified a range of factors that influence student engagement in PBL, broadly grouped into three categories. The first category involves instructional and interpersonal factors, particularly teacher behavior, autonomy support, and the quality of student–teacher interactions. When instructors provide clear guidance, constructive feedback, and emotionally supportive environments, students are more likely to invest effort and sustain interest in project tasks (Liu, 2024; Shin and Bolkan, 2021). The second category concerns structural and environmental factors, including task authenticity, resource availability, and institutional support structures that connect academic learning with practical contexts (Chang et al., 2024; Lombardi, 2007). The third category encompasses individual psychological factors such as self-efficacy, intrinsic motivation, and self-regulation, which mediate the effects of contextual conditions on engagement outcomes (Bandura, 1997; Shin, 2018). Although these three categories have been studied separately, less research has examined how they function together within a single integrated framework, particularly in interdisciplinary PBL settings.

However, PBL does not automatically produce meaningful engagement. Open-ended projects often involve ambiguity, heavy coordination demands, and sustained uncertainty. These features can create productive challenge, but they may also overwhelm students who lack confidence, resources, or clear guidance, and students in higher education PBL courses have indeed reported such difficulties, including the demanding and time-consuming nature of project work (Guo et al., 2020). In such contexts, engagement becomes a central condition for PBL success rather than a guaranteed consequence of the method itself. Student engagement is commonly understood as a multidimensional construct encompassing behavioral participation, emotional involvement, and cognitive investment in learning (Fredricks et al., 2004; Kahu, 2013; Kahu and Nelson, 2018; Xu et al., 2023; Zilvinskis et al., 2017). The present study focused on the behavioral and cognitive dimensions, which are the most observable and task-relevant components in PBL settings. In PBL, students must not only attend and complete tasks but also persist through uncertainty, regulate their learning strategies, and maintain interest throughout the project process.

Two contextual conditions are especially relevant to engagement in PBL. The first is teacher behavior and support (TBS), including autonomy support, constructive feedback, emotional encouragement, and clear instructional scaffolding. In student-centered project courses, teachers do not disappear; their role shifts toward designing structures, diagnosing obstacles, and helping students interpret setbacks as learning opportunities. Supportive teacher behavior satisfies students' needs for autonomy, competence, and relatedness, which are central to self-determination theory (SDT) (Jang et al., 2010; Niemiec and Ryan, 2009; Reeve and Cheon, 2021; Ryan, 2023; Ryan and Grolnick, 1986). Autonomy-supportive instruction predicts autonomous motivation and learning outcomes across diverse educational settings (Black and Deci, 2000), and recent classifications of motivational teaching behavior further distinguish the strategies teachers can use to support student motivation (Ahmadi et al., 2023). Recent studies have also shown that teacher–student relationships, intellectually stimulating instruction, and perceived teacher support are closely related to student engagement, often through motivational or psychosocial mediators (Gebre et al., 2025; Liu, 2024; Shin and Bolkan, 2021).

The second contextual condition is practical environment and support (PES). PBL is expected to connect academic learning with real-world problems, but this connection depends on practical resources, authentic tasks, institutional support, and opportunities for engagement with professional or industry contexts (Chang et al., 2024; Lombardi, 2007). Effective practical support requires not only well-designed project environments but also institutional structures that provide students with access to applied learning opportunities, professional partnerships, and relevant tools (Ferns et al., 2025). From the perspective of social cognitive theory (SCT), contextual support provides mastery experiences, vicarious experiences, and task-relevant resources that strengthen learners' beliefs in their ability to succeed (Bandura, 1997; Lent et al., 2000), and recent mixed-methods research confirms that mastery and vicarious experiences are among the most frequently reported sources of efficacy beliefs in educational settings (Gale et al., 2021). This perspective is also consistent with research on perceived organizational support, which indicates that individuals' perceptions of being valued by their environment contribute to their engagement and motivation (Eisenberger et al., 1986; Kurtessis et al., 2017). Thus, practical support may influence engagement by making the project environment more credible, navigable, and meaningful, while signaling to students that their learning efforts are institutionally valued.

Although TBS and PES are both forms of contextual support relevant to PBL, they are conceptually and functionally distinct. TBS primarily addresses students' psychological needs for autonomy, competence, and relatedness through interpersonal interactions, whereas PES addresses the structural and material conditions that make project work feasible and meaningful. Research on student engagement has increasingly recognized the importance of distinguishing between different types of support (Kahu and Nelson, 2018), yet existing PBL research in higher education has focused predominantly on student outcomes, leaving the mechanisms through which different forms of contextual support operate largely unexamined (Guo et al., 2020). Examining TBS and PES within the same model enables comparison of their relative contributions and identification of whether they operate through different psychological mechanisms.

The present study focuses on two psychological mechanisms through which contextual supports may affect engagement: self-efficacy and autonomous motivation. Self-efficacy refers to students' beliefs that they can organize and execute the actions required to complete learning tasks (Bandura, 1982). Prior educational psychology research has linked self-efficacy to self-regulated learning and classroom performance (Pintrich and de Groot, 1990). In PBL, self-efficacy is important because students must tolerate ambiguity, persist through failure, and coordinate complex tasks over time. Autonomous motivation refers to engaging in an activity because it is intrinsically interesting or because the activity has been internalized as personally important and valuable; it encompasses intrinsic motivation together with well-internalized forms of extrinsic motivation such as identified and integrated regulation (Gagné and Deci, 2005; Ryan and Deci, 2020). Cognitive evaluation theory further explains that events and contexts supporting perceived competence and autonomy enhance intrinsic motivation (Ryan, 1982), and recent research confirms that students who perceive their teachers as autonomy-supportive report higher intrinsic motivation and study effort in higher education settings (Torbergsen et al., 2023). Moreover, the internalization of autonomous motivation depends on the extent to which students experience social contexts that support their basic psychological needs (Williams and Deci, 1996).

Furthermore, we selected self-efficacy and autonomous motivation as mediators for three theoretical reasons. First, both constructs are central to the two theoretical frameworks informing this study: self-efficacy is the core mechanism in social cognitive theory (Bandura, 1997), while autonomous motivation, which subsumes intrinsic motivation and well-internalized forms of extrinsic motivation, is central to self-determination theory (Gagné and Deci, 2005; Ryan, 2023). Second, both SE and AM are malleable psychological states that can be influenced by contextual conditions such as teacher support and practical environment (Lent et al., 2000; Niemiec and Ryan, 2009). Third, both have been consistently linked to student engagement in higher education research, making them plausible proximal predictors of engagement (Fredricks et al., 2004; Kahu and Nelson, 2018). Indeed, recent research with Chinese undergraduates confirms that teacher support shapes engagement through autonomous motivation and self-efficacy (Guo et al., 2025). Other potential mediators, such as achievement goals, academic emotions, or social belonging, were not included because they are less directly tied to the specific contextual conditions of PBL or would have made the model excessively complex for the available sample.

Although previous studies have separately examined teacher support, contextual resources, self-efficacy, motivation, and engagement, less is known about how these factors operate together in interdisciplinary PBL courses in Chinese higher education. Treating support as a single construct may conceal the different functions of interpersonal support and practical environment support. Similarly, examining engagement without psychological mediators may leave unclear whether contextual conditions work mainly by changing students' internal readiness or by directly reducing barriers to participation.

Moreover, the context of Chinese higher education adds further relevance to this investigation. PBL has been increasingly adopted in Chinese universities as part of a broader push toward innovation-oriented talent cultivation, interdisciplinary curricula, and applied learning (Zhang and Ma, 2023). However, its implementation faces structural challenges that amplify the importance of both teacher support and practical environment support. Large class sizes, examination-oriented academic cultures, and relatively limited industry–university partnerships create conditions under which students need stronger interpersonal guidance and more clearly structured practical support to sustain deep engagement. At the same time, Chinese students in PBL contexts often report moderate levels of self-efficacy and intrinsic motivation, suggesting that these psychological resources are not automatically present and may depend on the quality of contextual support (Shin, 2018). Understanding how TBS and PES shape engagement through SE and AM in this specific context can inform the design of PBL curricula better aligned with the realities of Chinese higher education.

The present study responds to this gap by distinguishing TBS from PES and testing two theoretically grounded mediators within the same structural model. Specifically, this study proposes and tests an integrated PLS-SEM model in which TBS and PES predict student engagement directly and indirectly through self-efficacy and autonomous motivation. The study asks whether interpersonal and practical forms of support jointly relate to engagement and whether students' capability beliefs and autonomous interest are associated with these relationships.

## 2 Research model and hypotheses

Building on self-determination theory (Ryan, 2023) and social cognitive theory (Bandura, 1982), the proposed model specifies that two contextual forms of support, teacher behavior and support (TBS) and practical environment and support (PES), predict student engagement (SEng) through both direct and indirect pathways. The indirect pathways operate through two psychological mechanisms: self-efficacy (SE) and autonomous motivation (AM). The overall research model is depicted in Figure 1, with all 12 hypotheses detailed below.

**Figure 1:**
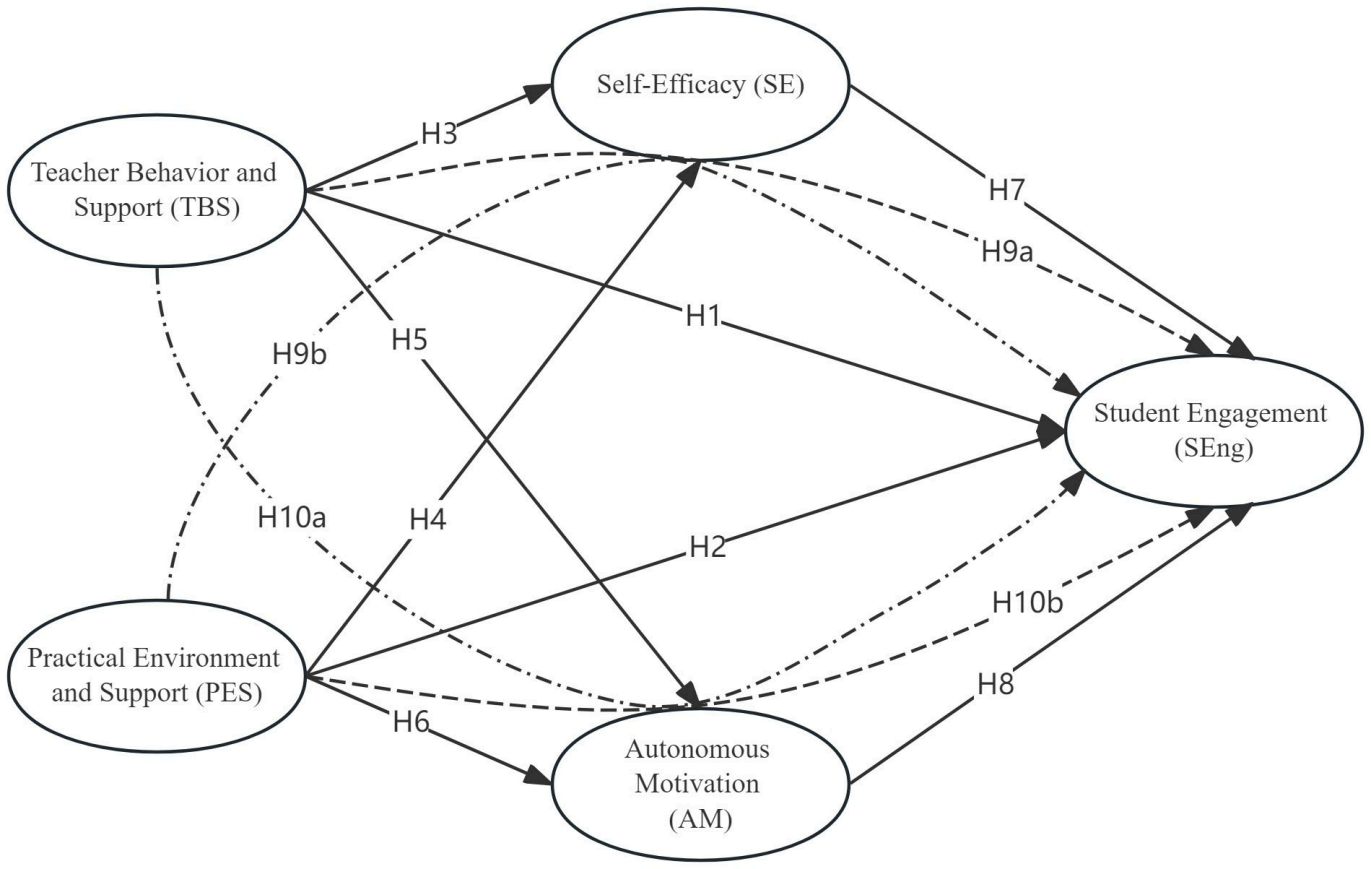
Conceptual model of the hypothesized relationships among TBS, PES, SE, AM, and student engagement (SEng).

### 2.1 Direct effects of contextual support on student engagement

According to SDT, social contexts that support individuals' basic psychological needs for autonomy, competence, and relatedness facilitate high-quality engagement, persistence, and wellbeing (Reeve and Cheon, 2021; Ryan, 2023). In PBL settings, TBS is a direct source of need support. When teachers provide autonomy-supportive instruction, constructive feedback, and emotional encouragement, they create conditions under which students feel both capable and self-directed in their project work. Empirical studies have consistently shown that perceived teacher support, including autonomy support and positive teacher–student relationships, is positively associated with student engagement across educational levels and learning contexts (Gebre et al., 2025; Liu, 2024). In PBL specifically, teacher guidance helps students navigate the ambiguity of open-ended projects, interpret setbacks as learning opportunities, and maintain commitment to project goals. Therefore, we propose:

**H1: TBS positively predicts SEng**.

Similarly, PES is expected to have a direct effect on student engagement. The quality and authenticity of the learning environment can directly influence how students participate in project work. When projects are embedded in authentic, real-world contexts and supported by adequate resources, tools, and institutional structures, students can more readily perceive the relevance of their work and sustain their involvement (Chang et al., 2024; Lombardi, 2007). Research on work-integrated learning and authentic assessment has shown that practical, contextually rich environments reduce the gap between academic learning and professional practice, thereby facilitating more meaningful engagement (Ferns et al., 2025). Moreover, when students perceive that their institution values their learning through the provision of practical support, they may reciprocate with higher levels of effort and engagement (Eisenberger et al., 1986). Thus, we propose:

**H2: PES positively predicts SEng**.

### 2.2 Effects of contextual support on self-efficacy

SCT posits that self-efficacy beliefs are shaped by four principal sources: mastery experiences, vicarious experiences, social persuasion, and physiological states (Bandura, 1997). Contextual conditions that provide these sources should therefore strengthen students' confidence in their ability to succeed. In PBL, TBS can enhance SE through several mechanisms. Teachers who offer constructive feedback and scaffolding help students interpret their performance as progress, creating mastery experiences that build confidence. Autonomy-supportive instruction also communicates respect for students' competence, serving as a form of social persuasion that reinforces efficacy beliefs (Black and Deci, 2000), and recent research confirms that perceived autonomy support positively predicts students' academic self-efficacy (Kingsford-Smith et al., 2024). Moreover, positive teacher–student relationships can reduce anxiety and create a psychologically safe environment in which students feel capable of taking on challenging tasks. These arguments lead to:

**H3: TBS positively predicts SE**.

PES is equally important for developing SE in PBL contexts. Authentic tasks that are appropriately challenging provide opportunities for students to experience success through direct engagement with real problems, thereby generating mastery experiences, which are consistently reported as the most influential source of efficacy beliefs (Gale et al., 2021). Access to relevant tools, resources, and professional contexts also exposes students to vicarious experiences through observations of how professionals or peers approach similar problems (Lent et al., 2000). When students have the practical means to execute project tasks, their confidence in their ability to complete those tasks is strengthened. Research on contextual supports and barriers in career development has demonstrated that environmental resources and supports significantly predict self-efficacy beliefs. Therefore:

**H4: PES positively predicts SE**.

### 2.3 Effects of contextual support on autonomous motivation

According to SDT's cognitive evaluation theory, events and contexts that enhance perceived competence and autonomy increase intrinsic motivation, whereas those that diminish perceived competence or autonomy undermine it (Deci and Ryan, 2000; Ryan, 1982). TBS, particularly autonomy-supportive teaching, directly addresses these conditions. Teachers who provide meaningful choices, rationale for tasks, and opportunities for self-direction support students' autonomy, while constructive feedback and appropriately structured guidance support their competence. Autonomy-supportive instruction has been shown to predict intrinsic motivation and autonomous forms of extrinsic motivation across diverse educational settings (Black and Deci, 2000; Niemiec and Ryan, 2009), and recent evidence confirms this pattern among university students, who report higher intrinsic motivation when they perceive their teachers as autonomy-supportive (Guo et al., 2025; Torbergsen et al., 2023). In PBL contexts, teacher support helps students maintain interest by framing project work as intrinsically valuable rather than externally mandated, conditions that support autonomous motivation. This leads to:

**H5: TBS positively predicts AM**.

PES also contributes to autonomous motivation by making project activities more interesting and meaningful. SDT suggests that tasks that are novel, challenging, and connected to individuals' interests are more likely to be experienced as intrinsically motivating (Ryan and Deci, 2020). PBL environments that provide authentic problems, real-world relevance, and tangible outcomes are inherently more interesting than decontextualized academic exercises, and this interest supports the development of autonomous motivation (Chang et al., 2024). Furthermore, having adequate resources and practical support reduces externally imposed barriers, allowing students to focus on the inherent satisfactions of project work. When the practical environment is well-structured and well-resourced, students can experience a greater sense of competence and autonomy, both antecedents of autonomous motivation. Thus:

**H6: PES positively predicts AM**.

### 2.4 Effects of psychological mediators on student engagement

Both SE and AM are well-established proximal predictors of student engagement. SCT maintains that individuals who believe they can successfully execute the actions required for a task are more likely to initiate effort, persist in the face of difficulty, and employ effective strategies (Bandura, 1997). In PBL, where tasks are complex, open-ended, and require sustained effort over time, SE is especially important. Students who doubt their ability to manage project demands are more likely to withdraw effort, avoid challenges, or disengage when obstacles arise. Empirical research on PBL has confirmed that self-efficacy is a significant predictor of both motivation and engagement in project environments (Guan et al., 2026; Shin, 2018). Accordingly:

**H7: SE positively predicts SEng**.

Autonomous motivation, which includes intrinsic interest and well-internalized forms of extrinsic motivation such as identified and integrated regulation (Gagné and Deci, 2005; Ryan and Deci, 2020), is another critical predictor of high-quality engagement. Students who are autonomously motivated invest effort because they find the learning activity itself rewarding or personally meaningful, which leads to deeper cognitive processing, greater persistence, and more positive affective experiences. In PBL contexts, autonomously motivated students are more likely to take ownership of their projects, explore problems beyond surface-level requirements, and remain engaged through iterative cycles of planning, execution, and revision. Research on student engagement in higher education has consistently found that autonomous motivation is positively associated with behavioral, emotional, and cognitive dimensions of engagement (Kahu and Nelson, 2018; Shin and Bolkan, 2021), and recent studies confirm this relationship across contemporary learning contexts, including online and AI-supported learning environments (Arugaslan, 2025; Xu et al., 2025). Therefore:

**H8: AM positively predicts SEng**.

### 2.5 Mediation hypotheses

The integrated model outlined above implies that TBS and PES influence SEng not only directly but also indirectly through SE and AM. The theoretical rationale for these indirect pathways is that contextual conditions operate partly by changing students' internal psychological states, which in turn shape their engagement. According to SCT, contextual resources and supports influence behavior through their effects on self-efficacy beliefs (Bandura, 1997). In PBL, supportive teacher behavior and practical environments provide the mastery experiences, social persuasion, and task-relevant resources that strengthen SE. Students with stronger SE are then more willing to invest sustained effort, persist through difficulties, and remain cognitively engaged in project tasks. This chain of effects represents a mediation pathway: TBS and PES → SE → SEng. We therefore propose:

**H9a and H9b: Self-efficacy mediates the relationships between (a) TBS and SEng and (b) PES and SEng**.

Similarly, SDT proposes that social-contextual factors supporting autonomy and competence enhance intrinsic motivation, which in turn promotes more active and sustained engagement (Ryan, 2023). In PBL, teachers who provide autonomy-supportive instruction and environments that offer authentic, meaningful tasks increase students' autonomous motivation. Autonomously motivated students are more likely to approach project work with interest, curiosity, and a sense of ownership, resulting in higher levels of engagement. Recent evidence confirms that perceived teacher support is associated with engagement through motivational mechanisms consistent with this pathway (Chen, 2025). This suggests a second mediation pathway: TBS and PES → AM → SEng. Thus:

**H10a and H10b: Autonomous motivation mediates the relationships between (a) TBS and SEng and (b) PES and SEng**.

The model includes 12 hypotheses: two direct effects from contextual support to engagement (H1–H2), four effects from contextual support to mediators (H3–H6), two direct effects from mediators to engagement (H7–H8), and four indirect effects representing the two mediation pathways (H9a, H9b, H10a, H10b). All 12 hypotheses are summarized in Tables 1, 2.

**Table 1:** Direct path coefficients.

Hypothesis	Path	β	*t*	*p*	*f^2^*	95% CI
H1	TBS → SEng	0.256	4.688	< 0.001	0.088	(0.153, 0.359)
H2	PES → SEng	0.209	4.851	< 0.001	0.050	(0.125, 0.293)
H3	TBS → SE	0.374	10.605	< 0.001	0.259	(0.307, 0.441)
H4	PES → SE	0.507	17.085	< 0.001	0.476	(0.449, 0.564)
H5	TBS → AM	0.436	11.206	< 0.001	0.366	(0.362, 0.511)
H6	PES → AM	0.469	12.407	< 0.001	0.424	(0.396, 0.543)
H7	SE → SEng	0.307	5.726	< 0.001	0.109	(0.204, 0.412)
H8	AM → SEng	0.175	3.920	< 0.001	0.034	(0.087, 0.261)

β, standardized path coefficient; *t*, t-statistic; *p*, significance level; *f*^2^, effect size (0.02 = small, 0.15 = medium, 0.35 = large); CI, bias-corrected 95% confidence interval. All paths were tested using 5,000 bootstrap resamples. All eight direct effect hypotheses (H1–H8) were supported.

**Table 2:** Indirect (mediation) effects.

Hypothesis	Path	β	*t*	*p*	95% CI	Mediation type
H9a	TBS → SE → SEng	0.115	4.902	< 0.001	(0.070, 0.163)	Partial
H9b	PES → SE → SEng	0.155	5.453	< 0.001	(0.101, 0.213)	Partial
H10a	TBS → AM → SEng	0.076	3.608	< 0.001	(0.036, 0.119)	Partial
H10b	PES → AM → SEng	0.082	3.757	< 0.001	(0.040, 0.125)	Partial

β, standardized indirect effect; *t*, t-statistic; *p*, significance level; CI, bias-corrected 95% confidence interval. Mediation is classified as partial because the direct effect of the predictor on the outcome remained significant after including the mediators. All four mediation hypotheses (H9a, H9b, H10a, H10b) were supported.

## 3 Materials and methods

### 3.1 Participants and procedure

Participants were 511 undergraduates (213 male, 298 female) enrolled in 21 interdisciplinary PBL courses at a university in eastern China, with course enrollments ranging from 10 to 42 students. The sample included undergraduates from four broad disciplinary fields (engineering, design, business, and education). The PBL courses integrated academic learning with practical problem solving and were designed to include collaborative project work, applied tasks, and opportunities to connect classroom learning with real-world problems. All participating courses were co-delivered by university instructors and industry mentors, so every undergraduate in the sample had access to the dual teaching team described in the Section 3.2.

Because undergraduates were nested within the 21 courses, we examined the independence assumption by computing intraclass correlation coefficients (ICCs) for the five constructs through one-way analyses of variance with course membership as the grouping factor. The ICCs were 0.000 for SEng, 0.003 for SE, 0.013 for TBS, 0.037 for AM, and 0.038 for PES, and the corresponding design effects [1 + (k – 1) × ICC, where *k* = 24.3 is the average course size] ranged from 1.00 to 1.89, all below the value of 2 below which clustering is commonly considered ignorable (Lai and Kwok, 2015). Although the *F* tests for AM (*F* = 1.924, *p* = 0.010) and PES (*F* = 1.972, *p* = 0.007) reached statistical significance, course membership accounted for at most 3.8% of the variance in any construct. Course-level nesting therefore does not appear to materially violate the independence assumption.

Data were collected near the end of the semester through an online questionnaire. Undergraduates evaluated their experiences of teacher behavior and support, practical environment and support, self-efficacy, autonomous motivation, and engagement in the PBL course. Participation was voluntary, and informed consent was obtained before the survey was completed. The questionnaire did not collect personally identifying information. Prior to the formal survey, the questionnaire was reviewed for clarity and course relevance. All 31 analyzed items were fully completed, with no missing values, and no responses were excluded after screening; because the survey was distributed online without a fixed quota, a conventional response rate cannot be computed.

### 3.2 Measures

All items were rated on a five-point Likert scale ranging from 1 (strongly disagree) to 5 (strongly agree). Five latent constructs were measured: teacher behavior and support (TBS, 8 items), practical environment and support (PES, 8 items), self-efficacy (SE, 5 items), autonomous motivation (AM, 5 items), and student engagement (SEng, 5 items). TBS items were developed with reference to the Learning Climate Questionnaire (Williams and Deci, 1996); SE items were adapted from the Self-Efficacy for Learning and Performance subscale of the Motivated Strategies for Learning Questionnaire (Pintrich et al., 1993); AM from the Intrinsic Motivation Inventory (Ryan, 1982); and SEng from the University Student Engagement Inventory (Maroco et al., 2016). PES items were developed based on the frameworks of perceived organizational support, social support, and authentic learning. All items were reviewed by two domain experts to ensure face and content validity prior to the formal survey.

**Teacher behavior and support (TBS)** was measured with eight items developed with reference to the Learning Climate Questionnaire (Williams and Deci, 1996), a standard instrument for measuring perceived teacher autonomy support within the self-determination theory framework, and modified to reflect the PBL context and the dual teaching team (university instructors and industry mentors) (Ahmadi et al., 2023; Almarwani et al., 2024; Jang et al., 2010; Prananto et al., 2025; Reeve and Cheon, 2021). The selected items captured autonomy support, feedback, and instructional guidance. A sample item is “The teaching team encouraged me to ask questions and express my ideas.” Cronbach's alpha was 0.899.

**Practical environment and support (PES)** was measured with eight items developed based on three theoretical frameworks: perceived organizational support (POS) (Eisenberger et al., 1986; Kurtessis et al., 2017), the Multidimensional Scale of Perceived Social Support (MSPSS) (Dambi et al., 2018; Pina et al., 2025; Zimet et al., 1988), and authentic learning (Ferns et al., 2025; Lombardi, 2007). Items assessed institutional resources, interpersonal support from different sources, and real-world learning design, with recent evidence supporting the relevance of these frameworks to student engagement in higher education (Chaudhry et al., 2024). Sample items include “The enterprise provided sufficient practical resources and platforms for my PBL project” and “In the PBL program, my classmates and I supported and helped each other.” Cronbach's alpha was 0.893.

**Self-efficacy (SE)** was measured with five items adapted from the Self-Efficacy for Learning and Performance subscale of the Motivated Strategies for Learning Questionnaire (Pintrich et al., 1993), reworded to reflect the PBL project context. Items captured students' confidence in achieving good results, mastering project-related skills, solving practical problems, and persisting through challenges. A sample item is “I'm confident I can master the skills and knowledge taught in this PBL course.” Cronbach's alpha was 0.820. The MSLQ continues to be widely applied in contemporary higher education research, including longitudinal studies of self-regulated learning (Esnaashari et al., 2025).

**Autonomous motivation (AM)** was measured with five items adapted from the Intrinsic Motivation Inventory (Ryan, 1982; Ryan et al., 1983). Following self-determination theory, autonomous motivation encompasses intrinsic motivation together with well-internalized forms of extrinsic motivation such as identified and integrated regulation (Gagné and Deci, 2005). The items therefore sampled the Interest/Enjoyment and Value/Usefulness subscales of the IMI, covering intrinsic interest and the internalization of the activity's personal value. A sample item is “I think PBL is a very interesting way of learning.” Cronbach's alpha was 0.800. The IMI has recently been adapted and validated for Chinese samples, supporting its cross-cultural applicability (Li et al., 2026).

**Student engagement (SEng)** was measured with five items adapted from the University Student Engagement Inventory (Maroco et al., 2016), which operationalizes the tripartite engagement framework (Fredricks et al., 2004). The items covered behavioral engagement (teacher discussion, peer interaction, collaborative work) and cognitive engagement (deep thinking, self-reflection) in the PBL context. A sample item is “During class, I think carefully about open-ended questions raised by the instructor.” Cronbach's alpha was 0.839. Recent meta-analytic evidence confirms that student engagement, measured through frameworks such as the USEI, is consistently associated with academic achievement and subjective wellbeing (Wong et al., 2024).

### 3.3 Data analysis

Data were analyzed using SmartPLS 4 (version 4.0.9.5) was developed by SmartPLS GmbH, based in Boenningstedt, Germany. A two-step PLS-SEM procedure was followed, consistent with common recommendations for evaluating reflective measurement models and structural relationships, and the guidelines for reporting PLS-SEM results were also followed (Hair et al., 2019). With 511 observations for a model of five constructs in which the most complex endogenous construct (SEng) is predicted by four structural paths (from TBS, PES, SE, and AM), the sample size satisfies the inverse square root rule for PLS-SEM (Kock and Hadaya, 2018): assuming a conservative minimum path coefficient of β = 0.15 at α = 0.05 with 80% power, this rule requires approximately 275 observations, which our sample exceeds by a wide margin. The sample also satisfies the widely used 10-times rule for PLS-SEM (minimum 40). After estimation, the weakest observed path (AM → SEng, β = 0.175) was stronger than the assumed minimum, and a *post hoc* power analysis in SmartPLS confirmed that the sample provides adequate statistical power for all hypothesized paths. First, the measurement model was assessed using indicator loadings, Cronbach's alpha, composite reliability, average variance extracted (AVE), Fornell–Larcker results, HTMT ratios, and VIF values (Fornell and Larcker, 1981; Henseler et al., 2015). Second, the structural model was evaluated using path coefficients, t statistics, p values, confidence intervals, *R*^2^ values, *f*^2^ effect sizes, and model fit indices. Bootstrapping with 5,000 resamples was used to test the significance of direct and indirect effects. Predictive relevance was assessed using the Stone–Geisser Q^2^ value obtained through blindfolding (Shmueli et al., 2019).

Because all constructs were measured using self-report survey data, common method bias (CMB) was carefully assessed. Procedural remedies included questionnaire anonymity, careful item wording to reduce evaluation apprehension, and reverse-coded items where appropriate. Statistically, three diagnostic approaches were applied. First, Harman's single-factor test showed that the first factor accounted for 32.6% of the total variance, below the 50% threshold (Podsakoff et al., 2003). Second, a full collinearity assessment was conducted following Kock (2015). The inner model VIF values were all well below the conservative 3.3 threshold: 2.075 for AM when predicting SEng, 2.021 for PES when predicting SEng, 1.992 for SE when predicting SEng, 1.732 for TBS when predicting SEng, 1.075 for TBS and 1.075 for PES when predicting AM, and 1.075 for TBS and 1.075 for PES when predicting SE. Third, following Lindell and Whitney (2001), we applied the marker-variable technique to adjust quantitatively for method variance, a technique that remains in current use in empirical research (Nikkhah et al., 2022; Saxena et al., 2024). Because the questionnaire did not include a marker variable—that is, an item measuring a construct theoretically unrelated to the substantive variables of the study—we adopted the conservative *post hoc* variant in which the smallest observed inter-construct correlation (*r* = 0.265, between TBS and PES) served as the proxy for the common method factor. Observed correlations were adjusted using the formula r_A = (r – r_s)/(1 – r_s). As shown in Table 3, all adjusted correlations remained statistically significant (*p* < 0.001). Together with the procedural remedies and the diagnostics described above, these results suggest that common method bias is unlikely to be a serious concern in this study.

**Table 3:** Inter-construct correlations adjusted for common method variance (Lindell–Whitney correction).

Construct pair	Observed *r*	Adjusted *r*	*t*	*p*
SEng–SE	0.654	0.529	14.07	< 0.001
PES–SE	0.606	0.464	11.82	< 0.001
AM–SEng	0.599	0.454	11.51	< 0.001
AM–PES	0.584	0.434	10.87	< 0.001
SEng–TBS	0.566	0.410	10.13	< 0.001
SEng–PES	0.565	0.408	10.09	< 0.001
AM–TBS	0.560	0.401	9.89	< 0.001
AM–SE	0.518	0.344	8.27	< 0.001
TBS–SE	0.508	0.331	7.90	< 0.001

The smallest observed inter-construct correlation (*r* = 0.265, TBS–PES) served as a conservative proxy for the common method factor (r_s). Adjusted correlations were computed as r_A = (r – r_s)/(1 – r_s) (Lindell and Whitney, 2001). All adjusted correlations remained statistically significant at *p* < 0.001 (*N* = 511), indicating that the construct relationships are not attributable to common method variance.

## 4 Results

### 4.1 Measurement model

The measurement model demonstrated acceptable reliability and convergent validity. Cronbach's alpha values ranged from 0.800 to 0.899, composite reliability (rho_c) ranged from 0.862 to 0.919, and AVE values ranged from 0.556 to 0.609 (Table 4). These values exceeded commonly used thresholds for internal consistency and convergent validity, and all standardized outer loadings exceeded the recommended threshold of 0.708, ranging from 0.712 to 0.859 (Table 5), indicating that the retained indicators adequately represented their constructs (Hair et al., 2020).

**Table 4:** Construct reliability and convergent validity.

Construct	Items	Loading range	α	rho_a	rho_c	AVE
Teacher Behavior and Support (TBS)	8	0.722–0.859	0.899	0.900	0.919	0.587
Practical Environment and Support (PES)	8	0.712–0.830	0.893	0.895	0.915	0.573
Self-Efficacy (SE)	5	0.751–0.776	0.820	0.821	0.874	0.582
Autonomous Motivation (AM)	5	0.715–0.784	0.800	0.803	0.862	0.556
Student Engagement (SEng)	5	0.753–0.840	0.839	0.842	0.886	0.609

α, Cronbach's alpha; rho_a, Dijkstra-Henseler's consistent reliability; rho_c, composite reliability; AVE, average variance extracted. All outer loadings exceeded 0.708. All constructs satisfied the thresholds of α ≥ 0.70, rho_c ≥ 0.70, and AVE ≥ 0.50 (Hair and Alamer, 2022; Hair et al., 2019).

**Table 5:** Standardized outer loadings and indicator-level VIF values.

Item	TBS	PES	SE	AM	SEng	VIF
TBS1	**0.779**					1.997
TBS2	**0.804**					2.238
TBS3	**0.729**					1.729
TBS4	**0.743**					1.826
TBS5	**0.741**					1.808
TBS6	**0.722**					1.710
TBS7	**0.742**					1.845
TBS8	**0.859**					2.827
PES1		**0.830**				2.467
PES2		**0.760**				1.834
PES3		**0.752**				1.782
PES4		**0.814**				2.268
PES5		**0.727**				1.726
PES6		**0.719**				1.700
PES7		**0.712**				1.624
PES8		**0.734**				1.750
SE1			**0.751**			1.563
SE2			**0.776**			1.705
SE3			**0.751**			1.584
SE4			**0.766**			1.665
SE5			**0.769**			1.615
AM1				**0.768**		1.555
AM2				**0.715**		1.457
AM3				**0.726**		1.473
AM4				**0.784**		1.660
AM5				**0.733**		1.483
SEng1					**0.840**	2.074
SEng2					**0.766**	1.661
SEng3					**0.753**	1.603
SEng4					**0.779**	1.726
SEng5					**0.761**	1.628

VIF, variance inflation factor. All 31 indicators loaded on their respective constructs with standardized loadings between 0.712 and 0.859, each exceeding the recommended threshold of 0.708 (Hair et al., 2019). Bold values indicate loadings exceeding this threshold. No indicator fell below the threshold, so no items were removed. All loadings were statistically significant at *p* < 0.001 based on 5,000 bootstrap resamples. All indicator-level VIF values were below 3.0, well below the conservative threshold of 5.0 (Hair et al., 2019), indicating no serious multicollinearity at the indicator level.

Discriminant validity was supported by HTMT and Fornell–Larcker results (Table 6). The highest HTMT value was 0.786 (between self-efficacy and student engagement), below the conservative 0.850 threshold, suggesting adequate separation among constructs. Fornell–Larcker results also showed that the square root of AVE for each construct was higher than its correlations with other constructs. All 31 indicator-level VIF values, reported item by item in Table 5, were below 3.0, well below the conservative threshold of 5.0 (Hair et al., 2019), indicating no serious multicollinearity concerns at the indicator level.

**Table 6:** Discriminant validity: HTMT ratios (above diagonal) and Fornell-Larcker criterion (below diagonal).

Construct	AM	SEng	PES	TBS	SE
AM	**(0.746)**	0.730	0.687	0.657	0.637
SEng	0.599	**(0.780)**	0.651	0.650	0.786
PES	0.584	0.565	**(0.757)**	0.294	0.706
TBS	0.560	0.566	0.265	**(0.766)**	0.591
SE	0.518	0.654	0.606	0.508	**(0.763)**

HTMT values are above the diagonal; Fornell-Larcker values (construct correlations) are below the diagonal. Bold diagonal values are the square root of AVE. All HTMT values are below the conservative threshold of 0.850, and all √AVE values exceed the corresponding inter-construct correlations, supporting discriminant validity (Fornell and Larcker, 1981; Henseler et al., 2015).

### 4.2 Structural model

All hypothesized direct effects were significant (Table 1). The estimated structural model, with all standardized path coefficients, is presented in Figure 2. TBS significantly predicted SEng (β = 0.256, *t* = 4.688, *p* < 0.001), SE (β = 0.374, *t* = 10.605, *p* < 0.001), and AM (β = 0.436, *t* = 11.206, *p* < 0.001). PES also significantly predicted SEng (β = 0.209, *t* = 4.851, *p* < 0.001), SE (β = 0.507, *t* = 17.085, *p* < 0.001), and AM (β = 0.469, *t* = 12.407, *p* < 0.001). In turn, SE (β = 0.307, *t* = 5.726, *p* < 0.001) and AM (β = 0.175, *t* = 3.920, *p* < 0.001) significantly predicted SEng. These results support all eight direct effect hypotheses (H1–H8).

**Figure 2:**
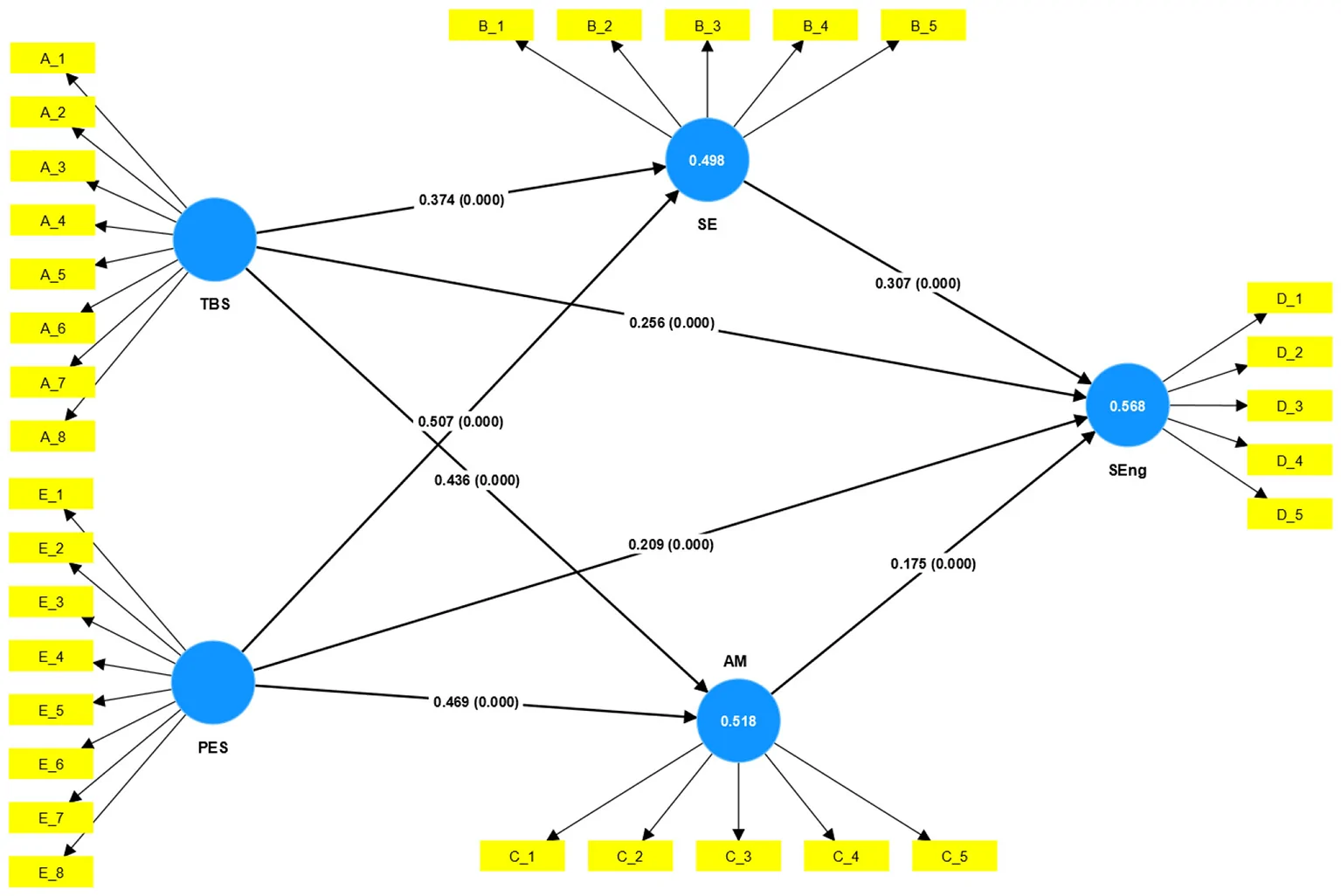
PLS-SEM results for the structural model. Standardized path coefficients are displayed; dashed paths indicate the four significant indirect effects.

Following Cohen's (1988) guidelines as applied in PLS-SEM (Hair and Alamer, 2022; Hair et al., 2019), *f*^2^ values of 0.02, 0.15, and 0.35 represent small, medium, and large effect sizes, respectively. Effect sizes indicated that PES had large effects on AM (*f*^2^ = 0.424) and SE (*f*^2^ = 0.476), and TBS had large effects on AM (*f*^2^ = 0.366) and medium-to-large effects on SE (*f*^2^ = 0.259). The direct effects of TBS and PES on SEng were smaller but still meaningful (*f*^2^ = 0.088 and 0.050, respectively). SE showed a larger effect size on SEng (*f*^2^ = 0.109) than AM (*f*^2^ = 0.034).

The model explained 51.8% of the variance in autonomous motivation, 49.8% of the variance in self-efficacy, and 56.8% of the variance in student engagement. Predictive relevance was assessed using the Stone–Geisser *Q*^2^ value obtained through blindfolding with an omission distance of 8. The *Q*^2^ values for autonomous motivation (*Q*^2^ = 0.284), self-efficacy (*Q*^2^ = 0.286), and student engagement (*Q*^2^ = 0.342) were all above zero, indicating satisfactory predictive relevance for all three endogenous constructs. The SRMR was 0.047 for both the saturated and estimated models, below the recommended 0.080 threshold (Hair et al., 2019; Henseler and Sarstedt, 2013), indicating acceptable model fit.

Although SE showed a numerically larger coefficient than AM in predicting SEng (β = 0.307 vs. 0.175), we formally tested whether this difference was statistically significant using a coefficient comparison based on the bootstrap standard errors (Hair et al., 2019). The coefficient difference was 0.132, with *t* = 1.878 (*p* = 0.060), and the bias-corrected 95% confidence intervals for the two coefficients overlapped [SE: (0.199, 0.412); AM: (0.085, 0.262)]. The difference therefore did not reach statistical significance, and the comparatively larger coefficient of SE should be interpreted as a descriptive pattern rather than a statistically demonstrated superiority.

As a robustness check, we re-estimated the model with PES modeled as a higher-order reflective construct with three first-order dimensions covering authentic learning, social support, and perceived organizational support. The second-order loadings were high for each first-order dimension: 0.888 for authentic learning, 0.914 for social support, and 0.920 for perceived organizational support, and the second-order AVE was 0.824, supporting the adequacy of treating PES as a single higher-order construct. The structural results were virtually unchanged relative to the first-order model used in the main analysis: PES → SE (β = 0.507), PES → AM (β = 0.470), PES → SEng (β = 0.206), TBS → SE (β = 0.377), TBS → AM (β = 0.438), TBS → SEng (β = 0.257), SE → SEng (β = 0.307), and AM → SEng (β = 0.176), with *R*^2^ for SEng remaining 0.568. These results indicate that the substantive findings, which are based on the first-order PES specification, are robust to an alternative, higher-order specification of the PES measurement model.

### 4.3 Mediation effects

The mediation results supported both self-efficacy and autonomous motivation as significant mediators (Table 2). PES had a significant indirect effect on SEng through AM [β = 0.082, *t* = 3.757, *p* < 0.001, 95% CI (0.040, 0.125)] and through SE [β = 0.155, *t* = 5.453, *p* < 0.001, 95% CI (0.101, 0.213)]. TBS also had a significant indirect effect on SEng through AM [β = 0.076, *t* = 3.608, *p* < 0.001, 95% CI (0.036, 0.119)] and through SE [β = 0.115, *t* = 4.902, *p* < 0.001, 95% CI (0.070, 0.163)]. Total indirect effects were significant for both PES (β = 0.238, *t* = 6.420, *p* < 0.001) and TBS (β = 0.191, *t* = 5.909, *p* < 0.001). Because the direct effects of TBS and PES on SEng remained significant after the mediators were included, the results indicate partial mediation.

## 5 Discussion

This study examined how teacher behavior and support (TBS) and practical environment and support (PES) relate to student engagement in project-based learning through self-efficacy (SE) and autonomous motivation (AM). The findings support the integrated model grounded in self-determination theory and social cognitive theory: both TBS and PES significantly predicted student engagement, and both effects were partially mediated by SE and AM. Together, the model explained 56.8% of the variance in student engagement. These results indicate that engagement in PBL is shaped by a combination of contextual and psychological conditions operating through distinct mechanisms.

### 5.1 TBS and student engagement

The first major finding is that TBS had significant direct (β = 0.256, *p* < 0.001) and indirect effects on student engagement, with the total effect (β = 0.447) substantially exceeding the direct effect. This confirms that teacher support relates to engagement both by directly facilitating participation and through students' psychological readiness. The significant indirect pathways through SE (β = 0.115, *p* < 0.001) and AM (β = 0.076, *p* < 0.001) indicate that approximately 43% of TBS's total effect on engagement is transmitted through these two mediators.

This finding is consistent with self-determination theory, which proposes that autonomy-supportive teaching and competence-building feedback satisfy students' basic psychological needs and thereby promote high-quality engagement (Reeve and Cheon, 2021; Ryan, 2023). The result also aligns with a substantial body of empirical evidence linking teacher–student relationships, perceived teacher support, and intellectually stimulating instruction to academic engagement and deep learning approaches (Gebre et al., 2025; Liu, 2024; Shin and Bolkan, 2021). Recent SDT-based studies have further confirmed these relationships across diverse educational contexts and populations, including Chinese university students (Yang et al., 2025), undergraduates in interdisciplinary PBL courses (Li et al., 2025), general higher education students (Orji et al., 2026), and cross-national PISA samples (Dagdeviren Ertaş and Bariş Pekmezci, 2025; Roorda et al., 2011). For example, Yang et al. (2025) demonstrated, using structural equation modeling with 593 Chinese university students, that socially supportive environments enhance learning motivation and engagement through the satisfaction of basic psychological needs, while Li et al. (2025) found, with 416 Chinese university students, that teacher autonomy support predicts academic engagement through the chain-mediating pathway of basic psychological needs satisfaction and autonomous motivation.

Furthermore, in PBL contexts, the role of teacher support extends beyond traditional instruction. Unlike lecture-based courses where teachers primarily transmit knowledge, PBL teachers serve as facilitators who diagnose obstacles, provide just-in-time feedback, and help students reinterpret setbacks as learning opportunities. The relatively large effect of TBS on AM (β = 0.436, *f*^2^ = 0.366) suggests that teacher support is especially important for sustaining students' autonomous interest in project work. This finding reinforces the argument that in student-centered learning environments, the teacher's role shifts from knowledge source to motivational architect.

### 5.2 PES and student engagement

The second major finding is that PES had both direct (β = 0.209, *p* < 0.001) and indirect effects on student engagement, with a total effect (β = 0.446) comparable to that of TBS. Notably, PES had large effects on SE (β = 0.507, *f*^2^ = 0.476) and AM (β = 0.469, *f*^2^ = 0.424), both exceeding the corresponding effects of TBS on these mediators. The indirect pathways through SE (β = 0.155, *p* < 0.001) and AM (β = 0.082, *p* < 0.001) together accounted for over 53% of PES's total effect.

These results demonstrate that practical environment and support are not peripheral features of PBL but central mechanisms through which PBL becomes credible and actionable for students. When learners have access to authentic tasks, usable resources, and meaningful contexts, they are more likely to believe that success is possible and to perceive project work as worthwhile. This aligns with Bandura's (1997) social cognitive theory, which identifies environmental resources as key sources of mastery and vicarious experiences that shape self-efficacy. It also supports research on authentic learning, which emphasizes that tasks embedded in real-world contexts sustain deeper engagement than decontextualized academic exercises (Chang et al., 2024; Lombardi, 2007). Taken together, these findings confirm that PES functions not merely as a contextual backdrop but as an active psychological resource that shapes students' capability beliefs and perceived task value.

Moreover, the particularly strong effects of PES on SE and AM deserve attention. The *f*^2^ values of 0.476 and 0.424 are considered large by conventional standards (Hair et al., 2019), suggesting that PES may be the more influential contextual condition for building students' psychological readiness in PBL. This pattern does not diminish the importance of TBS, which showed stronger direct effects on engagement itself, a medium-to-large effect on SE, and a large effect on AM. Rather, it suggests that TBS and PES play complementary roles: TBS is particularly important for directly shaping engagement behaviors through interpersonal interaction, while PES is especially potent for building the psychological resources (confidence and autonomous interest) that sustain engagement over time. This pattern may also reflect the specific characteristics of the sample: Chinese university students in interdisciplinary PBL courses may have limited prior exposure to practical, industry-connected learning environments, making the availability of authentic resources and institutional support especially impactful. When students encounter a well-resourced practical environment for the first time, the boost to their confidence and interest may be particularly pronounced.

### 5.3 Self-efficacy, autonomous motivation, and student engagement

The third important finding is that SE (β = 0.307, *f*^2^ = 0.109) showed a numerically larger direct effect on student engagement than AM (β = 0.175, *f*^2^ = 0.034), with an effect size more than three times that of AM. As reported in Section 4.2, however, the difference between the two coefficients did not reach statistical significance (*t* = 1.878, *p* = 0.060). The pattern is nonetheless descriptively consistent with the idea that the belief in one's capability to succeed in PBL tasks is closely associated with engagement, and that confidence may matter alongside interest rather than being redundant with it.

This pattern is theoretically meaningful in PBL contexts. While autonomous motivation is important for initiating engagement, complex project work requires sustained effort over extended periods, during which initial interest may fluctuate. Self-efficacy, by contrast, provides a more stable resource that helps students persist when tasks become ambiguous, solutions are incomplete, and feedback requires iteration. Previous PBL research has similarly found that self-efficacy plays a critical role in sustaining motivation and engagement in project environments (Guan et al., 2026; Shin, 2018), and recent work on college students has confirmed a close connection between academic self-efficacy and learning engagement in Chinese higher education (Wang and Zhang, 2024). Extending this evidence to broader higher education contexts, Faro et al. (2025) found, using SEM with 620 undergraduates, that self-efficacy significantly predicts student engagement, with self-efficacy, teacher support, and technology support collectively explaining 69% of the variance in engagement. Chun et al. (2025), employing PLS-SEM with 385 Chinese college students, further confirmed the mediating role of self-efficacy in transmitting the effects of cognitive and motivational factors on learning outcomes. In a large-sample study of 910 Chinese university students, He and Wen (2025) similarly found that self-efficacy and learning motivation mediated the relationship between perceived teacher support and online learning engagement, underscoring the robustness of the motivational mediation pathway across different learning modalities. Ilter (2026) demonstrated that academic self-efficacy is a significant predictor of academic engagement in higher education, with the relationship strengthened by perceived instructor support. Qi et al. (2024), employing meta-analytic path analysis, further established that perceived feedback influences academic performance sequentially through motivation and self-efficacy, providing meta-analytic support for the causal ordering assumed in our mediation model. Recent SDT-based studies provide further convergent evidence: Chen (2025) showed that perceived teacher support predicts student engagement through motivational mediation in AI-based learning contexts, Xu et al. (2025) found that personalized feedback enhances learning engagement through self-efficacy, and Dong (2025) confirmed the mediating role of digital self-efficacy in the relationship between learning engagement and motivation. These converging findings across different educational contexts reinforce the centrality of self-efficacy as a central predictor of student engagement.

The smaller but still significant effect of AM on engagement suggests that autonomous motivation operates alongside self-efficacy rather than being redundant with it. Students who find PBL activities inherently interesting are more likely to invest cognitive effort, explore beyond surface requirements, and remain engaged through iterative cycles of planning and revision. The partial mediation pattern also indicates that AM plays an important indirect role: contextual support is associated with stronger AM, which in turn relates to engagement. The key implication is that PBL design should deliberately cultivate both confidence and interest, rather than assuming that one automatically produces the other.

### 5.4 The partial mediation pattern

The fourth finding concerns the partial mediation pattern itself. All four specific indirect pathways (TBS → SE → SEng, TBS → AM → SEng, PES → SE → SEng, PES → AM → SEng) were significant, confirming that SE and AM serve as partial mediators of the relationships between contextual support and engagement. This aligns with recent PLS-SEM research by Arugaslan (2025), which similarly found that intrinsic motivation mediates the relationship between self-efficacy and student engagement, underscoring the dual pathway through which capability beliefs and autonomous interest jointly relate to engagement. The centrality of self-efficacy as a mediating mechanism is further supported by Akçay (2025), who demonstrated in a SEM study of 545 university students that academic self-efficacy partially mediates the relationship between learning competencies and learning outcomes. However, the direct effects of TBS and PES on engagement remained significant after accounting for the mediators, indicating that contextual support affects engagement through additional mechanisms beyond SE and AM.

This partial mediation pattern is theoretically important because it reveals that contextual support has both psychological and practical functions. On one hand, TBS and PES are associated with stronger engagement alongside students' confidence and autonomous interest, consistent with the mediation hypotheses derived from SDT and SCT. On the other hand, they also facilitate engagement by reducing practical barriers to participation. For example, clear instructional guidance directly enables students to navigate project tasks more effectively, well-equipped practical environments directly reduce logistical obstacles, and institutional support structures directly make project work more feasible. These direct effects would not be captured by psychological mediators alone.

However, the partial mediation pattern observed here may not hold universally across all PBL contexts. A recent systematic review by Prananto et al. (2025), synthesizing 13 higher education studies on teacher support and engagement, documented that the mediation pattern varies considerably across studies: some found full mediation through psychological needs or self-efficacy, others found partial mediation, and a few reported no mediation effect for certain pathways. This heterogeneity suggests that the relative strength of direct vs. indirect pathways depends on contextual moderators such as disciplinary field, project duration, group size, and the specific dimension of engagement measured. For instance, in highly structured projects where task ambiguity is low, the direct practical function of teacher guidance may be less critical, and psychological mechanisms may play a relatively larger role. Conversely, in interdisciplinary projects with high coordination demands, the direct enabling function of institutional support may be indispensable regardless of students' psychological readiness. Future research should test these boundary conditions systematically to establish when partial mediation holds and when alternative mediation patterns emerge.

### 5.5 Theoretical implications

This study makes three theoretical contributions to the PBL and student engagement literature. First, by integrating self-determination theory and social cognitive theory within a single empirical model, the study demonstrates that the two frameworks are complementary rather than competing when applied to PBL contexts. Self-determination theory explains why contextual support, particularly teacher autonomy support and meaningful tasks, fosters autonomous motivation and intrinsic interest (Ryan, 2023). Social cognitive theory explains why the same contextual conditions, particularly practical resources and authentic tasks, strengthen students' self-efficacy beliefs through mastery and vicarious experiences (Bandura, 1997). Tested separately, each theory would offer an incomplete account of how PBL engagement is sustained. Tested jointly, the model reveals that contextual support in PBL operates through dual psychological pathways (confidence and autonomous interest) rather than a single motivational channel. This dual-pathway perspective is consistent with recent evidence that self-determination and self-efficacy jointly shape student engagement and academic outcomes (Assefa et al., 2025; Yang et al., 2025), and suggests that future PBL research should adopt integrated theoretical frameworks rather than single-theory approaches.

Second, the study contributes a more differentiated conceptualization of contextual support by distinguishing TBS from PES. Existing PBL research has predominantly examined student outcomes rather than the distinct functions of different support types, leaving the comparative mechanisms of interpersonal vs. structural support largely unexplored (Guo et al., 2020). Our results show that TBS and PES have distinct functional profiles: PES is particularly important for building SE and AM (large effect sizes), while TBS has relatively stronger direct effects on engagement itself. This distinction has implications for both theory and practice, as it suggests that interpersonal and structural supports serve partially overlapping but partially distinct functions in fostering engagement. Conceptualizing support as a unidimensional construct would obscure these differential effects and may lead to underspecified theoretical models that cannot explain why some interventions succeed while others fail.

Third, the finding that self-efficacy showed a numerically larger coefficient than autonomous motivation in predicting engagement contributes to ongoing debates about the relative importance of different psychological mechanisms in complex learning environments. While both constructs matter, the comparatively larger coefficient of SE is descriptively consistent with the idea that in PBL contexts, characterized by ambiguity, sustained effort, and iterative problem solving, coping confidence may be a more critical psychological resource than task interest, although the difference was not statistically significant. This does not diminish the role of autonomous motivation; rather, it suggests that confidence and interest operate at different points in the engagement process. Autonomous motivation may be more important for initiating engagement, while self-efficacy may be more critical for sustaining it through the challenges that inevitably arise in project work. This interpretation extends prior research emphasizing the role of self-efficacy in self-regulated learning and academic performance (Pintrich and de Groot, 1990) and suggests that its importance may be amplified in project-based settings where uncertainty and coordination demands are high.

### 5.6 Practical implications

Interventions aimed at improving PBL engagement should address both psychological and structural pathways simultaneously. Efforts focused exclusively on building students' confidence or interest, while valuable, may be insufficient if the practical environment remains poorly structured or if teacher guidance is unclear. Conversely, improving the practical environment without attending to students' psychological readiness may also yield limited results. A dual approach that combines psychological support with structural support appears to be the most effective strategy.

More specifically, the findings suggest three practical directions for PBL course design. First, teachers should provide autonomy-supportive structure: students need meaningful choice, but they also need clear milestones, criteria, and formative feedback that help them build confidence in their ability to succeed. The strong path from TBS to SE (β = 0.374) underscores that teacher support is not only about creating a positive climate but is closely tied to students' capability beliefs through scaffolding and constructive feedback. Second, universities should invest in practical environments that make project work authentic. The large effects of PES on both SE and AM indicate that access to real problems, appropriate tools, and external partnerships is not optional but essential for PBL effectiveness. Institutions should prioritize the development of industry partnerships, applied learning spaces, and resource infrastructure that support interdisciplinary project work. Third, PBL curricula should deliberately cultivate self-efficacy through achievable sub-goals, visible progress tracking, peer modeling of successful project strategies, and feedback that helps students understand how to improve. Because SE showed a numerically larger coefficient than AM, although the difference was not statistically significant, interventions that specifically target students' confidence in managing project demands may be particularly valuable for engagement.

### 5.7 Limitations and future research

Several limitations should be acknowledged. First, the cross-sectional design limits causal inference. While the theoretical model assumes that contextual support precedes psychological states and engagement, the data are correlational. However, the hypothesized causal ordering is grounded in well-established longitudinal and experimental evidence from SDT and SCT, and our PLS-SEM results are consistent with this ordering. Future studies could use longitudinal designs to examine how TBS, PES, SE, AM, and engagement change across different stages of a project, or experimental designs to test causal effects by manipulating specific aspects of teacher behavior or practical support. Second, the study relied on self-report survey data, which may be affected by common method bias and social desirability. Although the CMB assessment suggested this is not a major concern—the Harman single-factor test yielded 32.6%, well below the 50% threshold, all inner model VIF values were below 3.3, and the marker-variable adjustment of Lindell and Whitney (2001) left all adjusted inter-construct correlations statistically significant (Table 3)—these diagnostics cannot entirely rule out method bias, because all variables were measured from the same source at the same time. Future research could incorporate multiple data sources such as behavioral logs, classroom observation, peer assessment, or performance outcomes to triangulate the findings. Third, the sample came from a single university in eastern China, which limits generalizability. Nevertheless, the consistency of our findings with prior multi-site and cross-national research (Chang et al., 2024; Roorda et al., 2011) suggests that the observed relationships are not idiosyncratic to this particular setting. Future studies should test the model across institutions, disciplines, and cultural settings to establish the boundary conditions of the proposed relationships. Fourth, the measurement of TBS and PES, while psychometrically adequate, could be refined in future research. The current study assessed TBS as a broad construct encompassing autonomy support, feedback, and emotional encouragement; future work could examine whether specific aspects of TBS (e.g., autonomy support vs. instructional scaffolding) have differential effects on SE and AM. Similarly, PES could be further differentiated into resource availability, task authenticity, and institutional support to identify which aspects of the practical environment are most consequential for engagement.

In addition, although the ICC analyses in Section 3.1 indicated negligible course-level variance, students were nonetheless nested within courses and teaching teams. Future research with a larger number of courses could employ multilevel models to examine course-level moderators explicitly, such as disciplinary field or project structure, and to model cross-level interactions between course characteristics and individual-level psychological states.

Fifth, the autonomous motivation measure encompassed intrinsic interest and the internalization of the activity's personal value, which were combined into a single construct. While structural equation modeling permits this treatment when items cohere adequately (α = 0.800; AVE = 0.556), future research may benefit from a more differentiated approach that captures distinct motivational subtypes separately, such as using the full IMI or the Self-Regulation Questionnaire to measure autonomous and controlled motivation independently. Sixth, the student engagement measure focused on behavioral and cognitive dimensions but did not include a separate emotional engagement subscale. While this aligns with the emphasis on observable participation and self-regulation in the PBL context, the tripartite framework (Fredricks et al., 2004) suggests that emotional engagement (feelings of belonging, interest, and identification with the learning community) may operate through distinct mechanisms not captured in the present study. Future research could incorporate the full USEI or other instruments that include emotional engagement items to test whether this dimension adds unique explanatory power beyond behavioral and cognitive engagement in PBL settings.

## 6 Conclusion

This study demonstrates that teacher behavior and support, together with practical environment and support, are both important for relating to student engagement in project-based learning. Their associations with engagement operate directly and indirectly through self-efficacy and autonomous motivation. Among the two mediators, self-efficacy showed a numerically larger coefficient in relation to engagement, although the difference did not reach statistical significance, suggesting that confidence in managing project demands is especially important in complex PBL environments. Effective PBL design should therefore combine supportive teaching, authentic practical environments, and deliberate strategies for building students' confidence and autonomous motivation.

These findings make three contributions to the literature. First, by integrating SDT and SCT within a single model, the study reveals that contextual support in PBL operates through dual psychological pathways rather than a single motivational channel. Second, by distinguishing teacher support from practical environment support, the study demonstrates that interpersonal and structural supports serve distinct functions. Third, the finding that self-efficacy's coefficient exceeded that of autonomous motivation, though not significantly, highlights the importance of cultivating students' capability beliefs in project-based settings. Together, these insights provide a foundation for designing more effective PBL environments that address both the interpersonal and structural conditions necessary for sustained student engagement.

## Data Availability

The original contributions presented in the study are included in the article/supplementary material, further inquiries can be directed to the corresponding author.
